# Secular trend in age at menarche in Egypt: a national study

**DOI:** 10.1007/s00431-026-07465-6

**Published:** 2026-11-03

**Authors:** Mohamed Abdelghafar Hussein, Eman M. Meatty, Elsayed A. Eid, Ahmed Attef, Mohamed Gad, Mostafa Awad Zaki, Belal Abdelhamid, Engy Ashraf, Abdel-Hady El-Gilany

**Affiliations:** 1 Department of Pediatrics, Faculty of Medicine, Kafrelsheikh University, Kafrelsheikh, Egypt; 2 Department of Pediatrics, Faculty of Medicine, Delta University for Science and Technology, Mansoura, Egypt; 3 Department of Internal Medicine, Faculty of Medicine, Delta University for Science and Technology, Mansoura, Egypt; 4 Faculty of Medicine, Delta University for Science & Technology, Mansoura, Egypt; 5 Department of Public Health, Faculty of Medicine, Mansoura University, Mansoura, Egypt

**Keywords:** Age at menarche, Secular trend, Puberty, Egyptian females, Birth cohorts, Pubertal timing

## Abstract

Age at menarche (AAM) is an important marker of female pubertal development and population health. Secular declines in AAM have been reported worldwide, but national data from Egypt remain limited. To evaluate secular trends in AAM among Egyptian females and identify factors associated with menarcheal timing. A national cross-sectional study was conducted among Egyptian females aged ≥ 10 years from multiple governorates. Data on AAM, residence, parental education, maternal employment, season of birth, birth order, and retrospectively self-reported weight status at menarche were collected using a structured questionnaire. Participants were classified into six birth cohorts to assess secular trends. Associations between AAM and potential determinants were analyzed using appropriate statistical methods. A total of 3,643 Egyptian females aged ≥ 10 years were included. A significant secular decline in AAM was observed across successive birth cohorts, with an approximately one-year reduction between the oldest and youngest cohorts (*p* < 0.001). Earlier AAM was significantly associated with urban residence and higher parental education. Significant regional variation was observed across governorates. In contrast, season of birth and maternal employment were not significantly associated with AAM.

*Conclusions*: Egyptian females have experienced a continuing decline in AAM, consistent with global trends toward earlier pubertal onset. Urbanization and socioeconomic factors were associated with earlier menarche. Ongoing monitoring of pubertal timing is warranted.

**Table:** 

**What is Known:** • *A secular decline in age at menarche has been reported globally.* • *Nutritional and socioeconomic transitions influence pubertal timing.*
**What is New:** • *This is among the first nationally distributed Egyptian studies evaluating secular trends in age at menarche.* • *Egyptian females demonstrated an approximately one-year decline in age at menarche across successive birth cohorts; earlier menarche was associated with urban residence and higher parental education in this Egyptian cohort.*

**Supplementary Information:**

The online version contains supplementary material available at https://doi.org/10.1007/s00431-026-07465-6.

## Introduction

Menarche refers to the first occurrence of menstruation and is associated with a variety of physical, psychological, and physiological changes in girls [1]. The age at which menarche occurs marks the initiation of menstrual function [2]. Menarche occurs as the final event in the sequence of pubertal development in girls, following thelarche, adrenarche, and the pubertal growth spurt [3].

Examining demographic patterns of menarche is important because this biological milestone generally indicates the beginning of the reproductive period, during which females become biologically capable of conceiving [4, 5]. Understanding the age at menarche in any context is important for patient education and can guide the clinical evaluation of patients to identify any deviation from normal expectations [6].

Several factors are known to influence the age at menarche (AAM), including genetic background, nutritional status, place of residence, geographic region, language group, race, climate, cultural environment, and socioeconomic status [7]. Previous studies have reported that girls with a higher body mass index (BMI), particularly those who are overweight or obese, tend to experience menarche earlier than girls with normal weight [8, 9]. Additionally, earlier menarche has been observed among girls living in urban settings and those from higher socioeconomic backgrounds compared with girls from lower socioeconomic groups [10]. Similarly, girls residing in urban areas generally attain menarche at a younger age than those living in rural regions [8].

A secular declining trend in the age of menarche has been reported in most developed and developing countries since the beginning of the twentieth century across the globe [11]. Many studies have been conducted to track this decline in recent times. A recent study by the Population Association of America noted a significant decline in the age at menarche worldwide, which was observed irrespective of socioeconomic status, race, or ethnicity. A downward trend from > 16 years in the mid-1800s to < 13 years by the 1980 s was reported [12]. Studies have also documented the trend towards earlier menarche in England [13], Israel [14], China [15, 16], India [17], Korea, Ghana [18], Mexico [19], Thailand [20], and the USA [21, 22]. In Europe, the menarcheal age decreased from 16 to 17 years in the mid-nineteenth century to 13 years in the mid-twentieth century [23].

Most studies on the secular trend in AAM were conducted in developing countries in previous decades. Only one study was conducted in Egypt about 15 years ago in a single district among adolescent schoolgirls and reported a mean AAM of 12.9 ± 1.03 years [24]. During this period, Egypt had experienced many social and economic changes, with changes in dietary habits, especially among the younger generation. However, no studies were carried out at the national level to highlight the secular trend in AAM across different age cohorts.

This study aims to estimate the mean AAM of different age cohorts at the national level to determine whether there is any secular trend in AAM.

## Population and methods

### Study locality and duration

This study was conducted at the national level across five governorates over a six-month period commencing in July 2025.

### Study design

A cross-sectional descriptive study with trend analysis.

### Target population

Women from different birth cohorts. Six 10-year birth cohorts were included: before 1965; 1965–1974; 1975–1984; 1985–1994; 1995–2004; and 2005–2014, corresponding to age cohorts ≥ 60, 59–50, 49–40, 39–30, 29–20, and 19–10 years, respectively.

### Eligibility criteria

Egyptian females aged 10 years or older who had experienced menarche and reported being able to recall their age at menarche.

### Sample size and sampling method

A multistage stratified cluster sampling method was used to ensure representation of women from the major regions of Egypt. Egypt was stratified into five regions: Greater Cairo, urban governorates, Lower Egypt, Upper Egypt, and the Frontier Governorates. One governorate was purposively selected from each region depending on the availability of trained local collaborators. Accordingly, Cairo (Greater Cairo) was selected as a highly urbanized metropolitan area; Alexandria as an urban governorate with no rural areas; Dakahlia (Lower Egypt) as a densely populated governorate; Qena (Upper Egypt) as a predominantly rural setting; and Matroh (Frontier Governorate) as a sparsely populated governorate.

Within each governorate, one district was selected, yielding a total of five districts. From each district, two localities were chosen—one city (district capital) and one village. In Alexandria, which is entirely urban, two urban districts were selected instead. Within each selected city or village, four clusters were identified to represent the northern, southern, eastern, and western areas. This yielded a total of 40 clusters (5 governorates × 1 district × 2 localities × 4 clusters = 40 clusters). Each cluster included at least 15 eligible females from each of the six age cohorts, giving a minimum target sample of 3,600 women (15 × 6 × 40 = 3,600). The first household in each cluster was randomly selected according to predefined randomization rules. In cases of refusal, the next eligible household was approached as a replacement. Local primary healthcare personnel and local community leaders assisted data collectors in identifying and selecting clusters and households. A total of 3,643 women were included in the study.

Data collected included governorate; date of birth (from national ID); education and work of participants; family residence; parental education and work; and retrospectively self-reported weight status at the time of menarche, categorized by the participant as normal weight, obese, or underweight.

### Data collection approach

The questionnaire was completed during a direct interview with women at their homes.

### Ethical consideration

The study protocol was approved by the Institutional Review Board of the Faculty of Medicine, Delta University, Egypt (IRB number: R 25-07-006) before data collection. All procedures followed the Declaration of Helsinki and national research ethics regulations. Informed consent was obtained from all participants. Participation was voluntary, and respondents were assured of data confidentiality. All data were collected anonymously, with no personally identifiable information recorded.

### Statistical analysis

Data were analyzed using the Statistical Package for the Social Sciences (SPSS), version 27. Categorical variables were presented as numbers and percentages. Age at menarche was found to be normally distributed according to the Shapiro test. Quantitative variables were presented as mean and SD. An unpaired t-test was used for comparisons between two groups, and one-way ANOVA was used for comparisons among multiple groups, with Bonferroni's post-hoc multiple comparisons. Two-way ANOVA was used to test interactions between age cohorts and different parameters. *p* ≤ 0.05 was considered statistically significant.

## Results

**Fig. 1:**
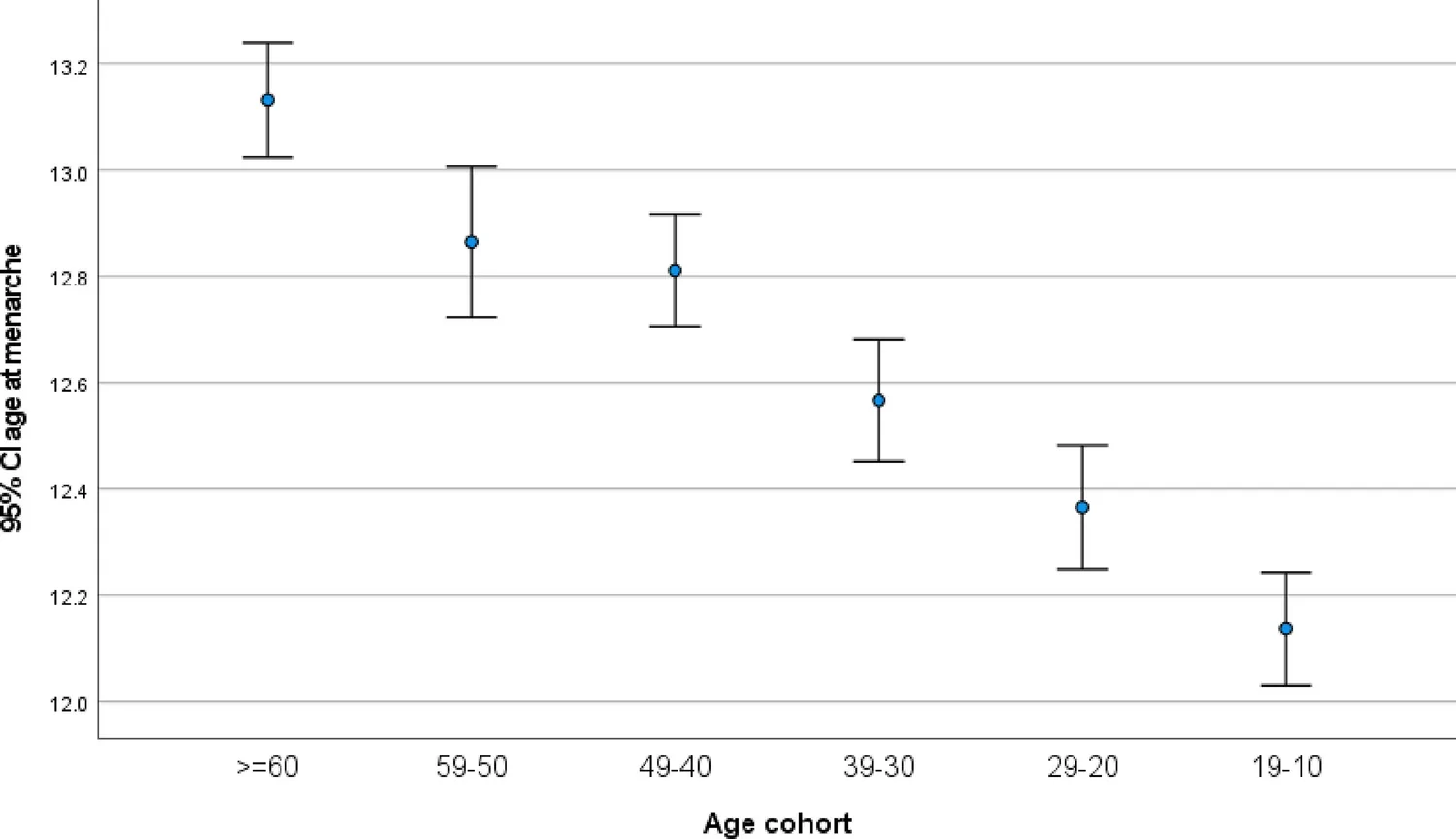
Error bar showing mean AAM (95% CI) in different age cohorts

Table 1 and Fig. 1 demonstrate the secular decline in mean age at menarche across successive birth cohorts.

**Table 1:** Secular trend in mean age at menarche in total sample

Birth cohort	Number (%)	Mean ± SD	95% CI of mean
(A) Before 1965 (≥ 60)	600 (16.47)	13.13 ± 1.35	(13.02–13.24)
(B) 1965–1974 (59–50)	601 (16.50)	12.87 ± 1.77	(12.72–13.01)
(C) 1975–1984 (49–40)	604 (16.58)	12.81 ± 1.33	(12.70–12.92)
(D) 1985–1994 (39–30)	607 (16.67)	12.57 ± 1.44	(12.45–12.68)
(E) 1995–2004 (29–20)	612 (16.80)	12.37 ± 1.47	(12.25–12.48)
(F) 2005–2014 (19–10)	619 (17.0)	12.14 ± 1.34	(12.03–12.24)
Total	3643 (100)	12.64 ± 1.49	(12.59–12.69)
Significance		F = 37.56,* p* ≤ 0.001	
Bonferroni’s post-hoc multiple comparisons (*p*-value)		A vs. B: 0.023	
		A vs. C:0.002	
		A vs. D: ≤ 0.001	
		A vs. E: ≤ 0.001	
		A vs. F: ≤ 0.001	
		B vs. C: 1.0	
		B vs. D: 0.006	
		B vs. E: ≤ 0.001	
		B vs. F: ≤ 0.001	
		C vs. D: 0.053	
		C vs. E: ≤ 0.001	
		C vs. F: ≤ 0.001	
		D vs. E: 0.244	
		D vs. F: ≤ 0.001	
		E vs. F: 0.089

Table 2 shows the variation in mean age at menarche across governorates and birth cohorts, with a significant governorate-by-cohort interaction.

**Table 2:** Secular trend in mean age at menarche by governorates

Age cohort	Governorates					*p* -value	
	Dakahlia (738, 20.3%)	Cairo (733, 20.1%)	Alexandria (732, 20.1%)	Qena (720, 19.8%)	Matrouh (720, 19.8%)	F-test	Two-way ANOVA
(A) Before 1965 (≥ 60)	13.94 ± 1.48 a,b,c,d	12.94 ± 1.53 a	12.56 ± 0.83 b,e,f	13.26 ± 1.38 c,e	13.02 ± 1.05 d,f	≤ 0.001	Governorate effect: F = 4.07, *p* = 0.01 Cohort effect: F = 2.57, *p* = 0.06 Governorate x cohort interaction: F = 15.25, *p* ≤ 0.001
(B) 1965–1974 (59–50)	13.21 ± 1.07 a,b	12.47 ± 1.61 a,c,d	11.79 ± 1.37 b,c,e,f	13.70 ± 1.182 d,e	13.16 ± 2.08 f	≤ 0.001	
(C) 1975–1984 (49–40)	12.81 ± 0.68 a,b	12.64 ± 1.40 c	11.63 ± 1.14 a,c,d,e	12.98 ± 1.81 d,f	13.91 ± 1.00 b,e,f	≤ 0.001	
(D) 1985–1994 (39–30)	12.33 ± 1.16 a	12.32 ± 1.59 B	12.36 ± 1.18 c	13.41 ± 1.49 a,b,c,d	12.42 ± 1.48 d	≤ 0.001	
(E) 1995–2004 (29–20)	12.5 ± 1.54 a	12.87 ± 1.83 a,b,c	12.28 ± 1.13	12.74 ± 1.10 B	12.51 ± 1.49 c	≤ 0.001	
(F) 2005–2014 (19–10)	11.38 ± 1.29 a,b,c,d	12.13 ± 1.64 a,e	12.20 ± 0.93 b,f	12.72 ± 1.13 C	12.14 ± 1.34 d,e,f	≤ 0.001	
Total	12.62 ± 1.47 a,b,c,d	12.49 ± 1.64 a,e,f,g	12.14 ± 1.15 b,e,h,i	13.11 ± 1.43 c,f,h	12.98 ± 1.51 d,g,i	≤ 0.001	
F-test (one-way ANOVA	≤ 0.001	≤ 0.001	≤ 0.001	≤ 0.001	≤ 0.001		
Bonferroni’s post-hoc multiple comparisons (*p*-value)							
A vs. B:	≤ 0.001	0.34	≤ 0.001	0.22	1.0		
A vs. C:	≤ 0.001	1.0	≤ 0.001	1.0	≤ 0.001		
A vs. D:	≤ 0.001	0.05	1.0	1.0	0.02		
A vs. E:	≤ 0.001	≤ 0.001	0.68	0.053	0.07		
A vs. F:	≤ 0.001	≤ 0.001	0.17	0.001	1.0		
B vs. C:	0.27	1.0	1.0	≤ 0.001	≤ 0.001		
B vs. D:	≤ 0.001	1.0	≤ 0.001	1.0	≤ 0.001		
B vs. E:	≤ 0.001	0.05	0.006	≤ 0.001	0.005		
B vs. F:	≤ 0.001	1.0	0.053	≤ 0.001	0.32		
C vs. D:	0.03	1.0	≤ 0.001	0.23	≤ 0.001		
C vs. E:	0.86	0.003	≤ 0.001	1.0	≤ 0.001		
C vs. F:	≤ 0.001	0.19	0.001	0.21	≤ 0.001		
D vs. E:	1.0	0.4	1.0	0.002	1.0		
D vs. F:	≤ 0.001	1.0	1.0	≤ 0.001	1.0		
E vs. F:	≤ 0.001	1.0	1.0	1.0	1.0		

a, b, c, d, e, f, g, h, i indicate significant pairwise differences between the corresponding governorates according to Bonferroni’s post-hoc multiple comparisons.

Table 3 demonstrates a significant association between paternal educational level and age at menarche, with lower menarcheal age observed among participants with higher paternal education.

**Table 3:** Secular trend in mean age at menarche by paternal education

Age cohort	paternal education				*P*-value	
	Illiterate (185,5.1%)	< secondary (1355,37.2%)	Secondary (1224,33.6%)	> secondary (879, 24.1%)	F-test	Two-way ANOVA
(A)Before 1965 (≥ 60)	14.18 ± 1.44 a,b,c	13.02 ± 1.58 a	13.25 ± 1.25 b	12.96 ± 1.05c	≤ 0.001	Father education effect: F = 7.43, *p* = 0.003 Cohort effect: F = 11.42, P ≤ 0.001 Father education X cohort interaction: F = 2.57, *p* ≤ 0.001
(B)1965–1974 (59–50)	13.88 ± 1.36 a	12.75 ± 1.76	13.04 ± 1.68	12.68 ± 1.89 a	0.02	
(C)1975–1984 (49–40)	13.26 ± 1.13	12.83 ± 1.33	12.83 ± 1.38	12.67 ± 1.29	0.20	
(D)1985–1994 (39–30)	13.08 ± 1.71	12.48 ± 1.54	12.56 ± 1.30	12.60 ± 1.34	0.13	
(E)1995–2004 (29–20)	13.74 ± 1.3 a,b,c	12.44 ± 1.43 a,d	12.17 ± 1.40b	11.87 ± 1.30 c,d	≤ 0.001	
(F)2005–2014 (19–10)	12.40 ± 1.47	12.20 ± 1.46	12.09 ± 1.29	12.10 ± 1.22	0.64	
Total	13.46 ± 1.53 a,b,c	12.62 ± 1.54 a,d	12.67 ± 1.44b	12.47 ± 1.22 c,d	≤ 0.001	
F-test (one-way ANOVA)	≤ 0.001	≤ 0.001	≤ 0.001	≤ 0.001		
Bonferroni’s post-hoc comparison (*p* -value)						
A vs. B:	1.0	0.88	1.0	1.0		
A vs. C:	0.43	1.0	0.024	1.0		
A vs. D:	0.08	0.002	≤ 0.001	0.47		
A vs. E:	1.0	0.001	≤ 0.001	≤ 0.001		
A vs. F:	0.002	≤ 0.001	≤ 0.001	≤ 0.001		
B vs. C:	1.0	1.0	1.0	1.0		
B vs. D:	0.93	0.86	0.007	1.0		
B vs. E:	1.0	0.44	≤ 0.001	≤ 0.001		
B vs. F:	0.035	0.004	≤ 0.001	0.002		
C vs. D:	1.0	0.18	0.82	1.0		
C vs. E:	1.0	0.08	≤ 0.001	≤ 0.001		
C vs. F:	0.71	≤ 0.001	≤ 0.001	0.005		
D vs. E:	0.45	1.0	0.12	≤ 0.001		
D vs. F:	1.0	0.79	0.008	0.03		
E vs. F:	0.007	1.0	1.0	1.0		

a, b, c, d indicate significant pairwise differences between paternal education categories within the same birth cohort according to Bonferroni’s post-hoc multiple comparisons. The reported *p* -values for A–F comparisons represent Bonferroni-adjusted pairwise comparisons between birth cohorts within each paternal education category.

Table 4 shows a significant association between maternal educational level and age at menarche, with earlier menarche observed among girls with higher maternal education.

**Table 4:** Secular trend in mean age at menarche by mother education

Age cohort	Mother education				*p* -value	
	Illiterate (187,5.1%)	< secondary (1588,43.0%)	Secondary (1206,33.1%)	> secondary (662, 18.2%)	F-test	Two-way ANOVA
(A)Before 1965 (≥ 60)	14.16 ± 1.50 a,b,c	13.04 ± 1.42 a	13.19 ± 1.32 b	13.06 ± 1.13 c	0.005	Mother education effect: F = 7.46, *p* = 0.003 Cohort effect: F = 9.32, *p* ≤ 0.001 Mother education X cohort interaction: F = 2.4, *p* = 0.002
(B) 1965–1974 (59–50)	13.53 ± 1.30	12.83 ± 1.80	12.96 ± 1.70	12.69 ± 1.86	0.26	
(C) 1975–1984 (49–40)	13.17 ± 1.04	12.90 ± 1.29	12.76 ± 1.38	12.53 ± 1.38	0.053	
(D) 1985–1994 (39–30)	13.21 ± 1.64 a,b	12.50 ± 1.43 a	12.42 ± 1.37 b	12.83 ± 1.49	0.004	
(E) 1995–2004 (29–20)	13.70 ± 1.38 a,b,c	12.39 ± 1.38 a,d,e	12.03 ± 1.36 b,d	11.95 ± 1.44 c,e	≤ 0.001	
(F) 2005–2014 (19–10)	12.95 ± 1.90 a,b	12.17 ± 1.37	12.09 ± 1.37 a	12.06 ± 1.13 b	0.049	
Total	13.48 ± 1.48 a,b,c	12.64 ± 1.48 a	12.59 ± 1.49 b	12.5 ± 1.47 c	≤ 0.001	
F-test (one-way ANOVA)	0.061	≤ 0.001	≤ 0.001	≤ 0.001		
Bonferroni’s post-hoc comparison (*p* -value)						
A vs. B:		1.0	≤ 0.001	0.64		
A vs. C:		1.0	0.034	0.13		
A vs. D:		≤ 0.001	≤ 0.001	1.0		
A vs. E:		≤ 0.001	≤ 0.001	≤ 0.001		
A vs. F:		≤ 0.001	≤ 0.001	≤ 0.001		
B vs. C:		1.0	1.0	1.0		
B vs. D:		0.12	0.002	1.0		
B vs. E:		0.007	≤ 0.001	0.002		
B vs. F:		≤ 0.001	≤ 0.001	0.005		
C vs. D:		0.016	0.24	1.0		
C vs. E:		≤ 0.001	≤ 0.001	0.07		
C vs. F:		≤ 0.001	≤ 0.001	0.20		
D vs. E:		1.0	0.15	≤ 0.001		
D vs. F:		0.14	0.27	0.001		
E vs. F:		1.0	1.0	1.0		

a, b, c indicate significant pairwise differences between the corresponding maternal education categories according to Bonferroni’s post-hoc multiple comparisons.

Table 5 shows a significant association between retrospectively self-reported weight status at menarche and age at menarche. Participants who reported being obese at menarche had a lower mean age at menarche than those who reported normal weight or underweight status (*p* < 0.001). This finding should be interpreted cautiously because weight status was retrospectively self-reported and was not based on contemporaneous anthropometric measurements.

**Table 5:** Secular trend in mean age at menarche by retrospectively self-reported weight status at menarche

Age cohort	Weight status at menarche			*p* -value	
	Normal* (1951,53.6%)	Obese* (857, 23.5%)	Underweight* (835, 22.9%)	F-test (ANOVA)	Two-way ANOVA
(A)Before 1965 (≥ 60)	13.41 ± 1.31 a,b	12.51 ± 1.50 a,c	12.99 ± 1.10 b,c	≤ 0.001	Weight-status effect: F = 40.58, *p* ≤ 0.001 Cohort effect: F = 27.32, *p* ≤ 0.001 Weight-status × cohort interaction: F = 3.16, *p* ≤ 0.001
(B)1965–1974 (59–50)	13.18 ± 1.71 a,b	12.41 ± 1.64 a	12.69 ± 1.89b	≤ 0.001	
(C)1975–1984 (49–40)	12.91 ± 1.19	12.67 ± 1.57	12.73 ± 1.37	0.16	
(D)1985–1994 (39–30)	12.67 ± 1.43	12.51 ± 1.55	12.42 ± 1.34	0.19	
(E)1995–2004 (29–20)	12.65 ± 1.38 a,b	11.99 ± 1.57 a	12.09 ± 1.43 b	≤ 0.001	
(F)2005–2014 (19–10)	12.19 ± 1.32 a	11.81 ± 1.31 a,b	12.31 ± 1.37 b	0.005	
Total	12.83 ± 1.45 a,b	12.31 ± 1.56 a,c	12.55 ± 1.47 b,c	≤ 0.001	
F-test (ANOVA)	≤ 0.001	≤ 0.001	≤ 0.001		
Bonferroni’s post-hoc comparison (*p* -value)					
A vs. B:	0.56	1.0	1.0		
A vs. C:	≤ 0.001	1.0	1.0		
A vs. D:	≤ 0.001	1.0	0.01		
A vs. E:	≤ 0.001	0.08	≤ 0.001		
A vs. F:	≤ 0.001	0.005	0.001		
B vs. C:	0.24	1.0	1.0		
B vs. D:	≤ 0.001	1.0	1.0		
B vs. E:	≤ 0.001	0.23	0.01		
B vs. F:	≤ 0.001	0.015	0.38		
C vs. D:	0.47	1.0	0.90		
C vs. E:	0.27	0.002	0.006		
C vs. F:	≤ 0.001	≤ 0.001	0.21		
D vs. E:	1.0	0.04	1.0		
D vs. F:	≤ 0.001	0.002	1.0		
E vs. F:	≤ 0.001	1.0	1.0		

a, b, c indicate significant pairwise differences between the corresponding weight-status categories according to Bonferroni’s post-hoc multiple comparisons.

*Weight status at menarche was retrospectively self-reported by participants and was not based on contemporaneous anthropometric measurements or BMI.

Additional analyses according to residence, father occupation, maternal employment, season of birth, and birth order are provided in Supplementary Tables S1-S5.

## Discussion

The present Egyptian national study demonstrates a significant secular decline in age at menarche (AAM) among Egyptian females, with an approximately one-year reduction across successive birth cohorts. This finding is consistent with the global trend toward earlier pubertal onset observed over recent decades.

This decline agrees with findings from several international populations. Studies from Europe and North America have documented progressive reductions in AAM, commonly attributed to improvements in nutrition, healthcare, and socioeconomic conditions [21, 22]. Similar trends have also been reported in Asia, including large-scale studies from China [7]. Recent global analyses further suggest that this decline continues, particularly in low- and middle-income countries undergoing rapid epidemiological and nutritional transition [23].

Within Egypt, the present findings provide important longitudinal insight compared with previous local data. El-Gilany [20] reported a mean AAM of approximately 12.9 years among schoolgirls in Mansoura in 2008. The lower estimates observed in the youngest cohort in the current study indicate that the decline in menarcheal age has continued over the past 15 years. This temporal shift may reflect broader changes accompanying Egypt’s epidemiological and nutritional transition, including urbanization, dietary changes, and improvements in living conditions [24–26].

The observed pattern is also consistent with findings from other Arab populations, where earlier menarche has increasingly been reported. Studies from Gulf countries, including Saudi Arabia and the United Arab Emirates, documented mean AAM values between 12 and 13 years, with evidence of a continuing decline attributed to rapid socioeconomic development and lifestyle transition [21, 22, 27].

Comparable trends have also been reported across Africa, although with regional variability. A study from Ghana [8] reported a mean AAM of approximately 13 years, with earlier menarche associated with urban residence and higher socioeconomic status. These findings are consistent with an association between urbanization, nutritional status, and pubertal timing. Collectively, these observations place Egypt within a transitional spectrum between traditional and industrialized patterns of pubertal development.

At the population level, the decline in AAM may also be explained by improvements in nutritional status and general health conditions. Adequate childhood nutrition is essential for activation of the hypothalamic–pituitary–gonadal axis and normal pubertal progression. Epidemiological evidence consistently shows that improved food security, reduced infectious disease burden, and better healthcare access are associated with earlier pubertal onset [21, 28–32]. Conversely, chronic illness and undernutrition are linked to delayed menarche.

From a biological perspective, nutritional status can influence pubertal timing through complex metabolic and hormonal pathways. Increased adiposity has been associated with elevated leptin, insulin, and insulin-like growth factor-1 levels, which may act as permissive signals for pubertal initiation. Evidence from other populations has also suggested that early-life nutritional exposures and rapid childhood weight gain may influence long-term pubertal trajectories [29]. However, these mechanisms should be considered in the context of the existing literature, as the present study did not assess childhood BMI or longitudinal nutritional exposures.

A key finding of the current study was the persistent urban–rural disparity, with urban girls consistently experiencing earlier menarche than rural girls. Similar observations have been widely reported internationally and may be related to differences in dietary patterns, physical activity, and environmental exposures. Urban environments are commonly associated with increased caloric intake, sedentary behavior, and greater exposure to endocrine-disrupting chemicals, all of which may accelerate pubertal onset [26], a pattern also observed in rapidly urbanizing Middle Eastern populations [27].

The association between higher parental education and earlier menarche further highlights the influence of socioeconomic factors on pubertal timing. Higher educational attainment is generally associated with improved nutrition, healthcare access, and living conditions, contributing to accelerated growth and maturation. Similar associations have been documented in African and Middle Eastern populations [10, 14, 33].

The present study also identified an association between earlier AAM and retrospectively self-reported weight status at menarche. Participants who reported being obese at menarche had a lower mean AAM than those reporting normal weight or underweight status. However, this finding should be interpreted cautiously because weight status was retrospectively self-reported and was not based on contemporaneous anthropometric measurements. Therefore, the present data do not allow reliable assessment of the contribution of objectively measured adiposity to pubertal timing or establishment of a causal relationship. The observed association is nevertheless consistent with the broader literature linking higher adiposity with earlier pubertal development [28].

In contrast, no significant relationship was identified between AAM and season of birth or maternal employment. These findings suggest that broader biological and socioeconomic determinants may exert stronger influences on pubertal timing than short-term environmental or household-level factors. Similar observations have been reported previously [22, 34, 35].

The study also demonstrated significant regional variation across governorates, likely reflecting differences in socioeconomic development, dietary patterns, healthcare access, and cultural practices. These findings emphasize the heterogeneity of pubertal timing within the Egyptian population and support the need for region-specific public health strategies.

Despite its strengths, including the large sample size and nationally distributed design, this study has several limitations. First, AAM was retrospectively self-reported and may therefore be subject to recall bias, particularly among older participants because of the longer interval between menarche and study participation. Although previous epidemiological studies have reported reasonable reliability of recalled menarcheal age [36, 37], recalled AAM was not independently validated against contemporaneous records in the present study. Second, weight status at menarche was also retrospectively self-reported and was not based on contemporaneous anthropometric measurements or BMI, which may have resulted in misclassification and reporting bias. Therefore, the observed association between self-reported weight status and AAM should be interpreted cautiously. Third, the cross-sectional design limits causal inference and relies on retrospective comparisons between birth cohorts rather than longitudinal follow-up. Finally, although efforts were made to ensure broad geographic representation, the purposive selection of governorates may limit the generalizability of the findings.

## Conclusion

This study provides national evidence of a continuing decline in age at menarche in Egypt, consistent with global, regional, and African trends. The findings highlight significant associations of urban residence and parental education with pubertal timing, while the association with retrospectively self-reported weight status should be interpreted cautiously. Given the potential long-term health implications of earlier menarche, including its associations with obesity and metabolic and reproductive health outcomes, these findings may have implications for pediatric healthcare and public health planning in Egypt and similar settings.

## Supplementary Information

Below is the link to the electronic supplementary material.

Supplementary file1 (DOCX 31 KB)

## Data Availability

The datasets generated and/or analyzed during the current study are not publicly available due to institutional and ethical restrictions but are available from the corresponding author on reasonable request.
