# Supplementary material for: Secular trend in age at menarche in Egypt: a national study
Source: Eur J Pediatr. 2026 Nov 3;185(11):914. doi: 10.1007/s00431-026-07465-6 (PMC13731495; doi:10.1007/s00431-026-07465-6)
Supplement: Supplementary file 1 [file 431_2026_7465_MOESM1_ESM.docx]

**Supplementary file**

**Secular trend in age at menarche in Egypt:**

**A national study**

**Authors: Mohamed Abdelghafar Hussein, Eman M. Meatty**, **Elsayed A. Eid,** **Ahmed Attef , Mohamed Gad, Mostafa awad zaki, Belal Abdelhamid , Engy Ashraf , Abdel-Hady El-Gilany**

Table S1 shows a significant association between residence and age at menarche, with urban participants consistently reporting a lower mean age at menarche than rural participants across birth cohorts. A significant cohort effect was also observed.

**Table S1: Secular trend in mean age at menarche by residence**

| Age cohort | Residence | | *p* -value | |
| --- | --- | --- | --- | --- |
|  | Rural (1511, 41.5%) | Urban (2132, 58.5%) | Independent t-test | Two-way ANOVA |
| (A) Before 1965 (≥60) | 13.62 ± 1.60 | 12.82 ± 1.06 | ≤0.001 | Residence effect: F=165.8, *p* ≤0.001  Cohort effect: F=40.35, *p* ≤0.001  Residence x cohort interaction: F=1.7, *p* =0.13 |
| (B) 1965-1974 (59-50) | 13.07 ± 1.38 | 12.19 ± 1.43 | ≤0.001 |  |
| (C) 1975-1984 (49-40) | 13.24 ± 1.25 | 12.68 ± 1.34 | ≤0.001 |  |
| (D) 1985-1994 (39-30) | 12.91 ± 1.58 | 12.31 ± 1.27 | ≤0.001 |  |
| (E) 1995-2004 (29-20) | 12.5 ± 1.70 | 11.93 ± 1.26 | ≤0.001 |  |
| (F) 2005-2014 (19-10) | 12.49 ± 1.53 | 12.16 ± 1.30 | 0.004 |  |
| Total | 13.00 ± 1.60 | 12.39 ± 1.36 | ≤0.001 |  |
| F-test (one-way ANOVA) | ≤0.001 | ≤0.001 |  |  |
| Bonferroni’s post-hoc comparison (*p* -value)  A vs. B:  A vs. C:  A vs. D:  A vs. E:  A vs. F:  B vs. C:  B vs. D:  B vs. E:  B vs. F:  C vs. D:  C vs. E:  C vs. F:  D vs. E:  D vs. F:  E vs. F: | 0.30  0.017  ≤0.001  ≤0.001  ≤0.001  1.0  0.06  ≤0.001  ≤0.001  1.0  0.02  ≤0.001  1.0  0.003  0.51 | 0.02  0.42  ≤0.001  ≤0.001  ≤0.001  1.0  1.0  0.01  ≤0.001  0.04  ≤0.001  ≤0.001  1.0  0.01  1.0 |  |  |

Table S2 demonstrates a significant association between paternal occupation and age at menarche, with differences in mean age at menarche observed across occupational categories.

**Table S2: Secular trend in mean age at menarche by father occupation**

| Age cohort | father occupation | | | | *p* -value | |
| --- | --- | --- | --- | --- | --- | --- |
|  | Employee  (1451,39.8%) | Farmer/Manual worker (1377, 37.8%) | Not working (248, 6.8%) | Others* (567, 15.8%) | F-test (ANOVA) | Two-way ANOVA |
| (A) Before 1965 (≥60) | 13.15±1.47 | 13.25 ± 1.44 | 12.67 ± 0.87 | 13.02 ± 0.91 | 0.07 | Father occupation effect: F1.52=, *p* =0.25  Cohort effect: F=7.07, *p* ≤0.001  Father occupation x cohort interaction: F3.10=, *p* ≤0.001 |
| (B) 1965-1974 (59-50) | 12.95±1.72 | 12.82 ± 1.73 | 12.17 ± 1.51 | 13.04 ± 2.04 | 0.07 |  |
| (C) 1975-1984 (49-40) | 12.81±1.40  a,b | 12.69 ± 1.22  c,d | 11.8 ± 1.20  a,c,e | 13.31 ± 1.24  b,d,e | ≤0.001 |  |
| (D) 1985-1994 (39-30) | 12.52±1.41 | 12.64 ± 1.47 | 12.63 ± 1.48 | 12.45 ± 1.45 | 0.68 |  |
| (E) 1995-2004 (29-20) | 12.35±1.66 | 12.52 ± 1.43a | 12.41 ± 0.90 | 11.97 ± 1.23 a | 0.046 |  |
| (F) 2005-2014 (19-10) | 12.01 ± 1.42 | 12.44 ± 1.35 | 12.18 ± 0.89  a,b | 12.21 ± 1.24  b | 0.26 |  |
| Total | 12.62 ± 1.56 | 12.7 ± 1.48a | 12.36 ± 1.17 | 12.69 ± 1.45 | 0.007 |  |
| F-test (ANOVA) | ≤0.001 | ≤0.001 | 0.02 | ≤0.001 |  |  |
| Bonferroni’s post-hoc comparison (*p* -value)  A vs. B:  A vs. C:  A vs. D:  A vs. E:  A vs. F:  B vs. C:  B vs. D:  B vs. E:  B vs. F:  C vs. D:  C vs. E:  C vs. F:  D vs. E:  D vs. F:  E vs. F: | 1.0  0.24  ≤0.001  ≤0.001  ≤0.001  1.0  0.03  ≤0.001  ≤0.001  0.47  0.009  ≤0.001  1.0  0.003  0.19 | 0.019  0.001  ≤0.001  ≤0.001  ≤0.001  ≤0.001  1.0  1.0  ≤0.001  1.0  1.0  0.02  1.0  0.052  0.073 | 0.98  0.053  1.0  1.0  0.94  1.0  1.0  1.0  1.0  0.06  0.39  1.0  1.0  1.0  1.0 | 1.0  1.0  0.08  ≤0.001  ≤0.001  1.0  0.08  ≤0.001  ≤0.001  ≤0.001  ≤0.001  ≤0.001  0.47  1.0  1.0 |  |  |

a,b,c,d,e indicate significant pairwise differences between the corresponding paternal occupation categories according to Bonferroni’s post-hoc multiple comparisons

*Business & trades

Table S3 shows no significant association between maternal employment status and age at menarche, although a significant birth cohort effect was observed.

**Table S3: Secular trend in mean age at menarche by mother work**

| Age cohort | Mother work | | *p* -value | |
| --- | --- | --- | --- | --- |
|  | Not working (2270, 62.3%) | Working  (1373 37.7%) | Independent t-test | Two-way ANOVA |
| (A) Before 1965 (≥60) | 12.97 ± 1.23 | 13.37±1.48 | ≤0.001 | Mother work effect: F=0.26, *p* =0.63  Cohort effect: F=7.39, *p* =0.023  Mother work x cohort interaction: F=4.83, *p* ≤0.001 |
| (B) 1965-1974 (59-50) | 12.87 ± 1.86 | 12.85 ± 1.62 | 0.90 |  |
| (C) 1975-1984 (49-40) | 12.90 ± 1.35 | 12.65 ± 1.28 | 0.024 |  |
| (D) 1985-1994 (39-30) | 12.64 ± 1.50 | 12.46 ± 1.35 | 0.13 |  |
| (E) 1995-2004 (29-20) | 12.24 ± 1.41 | 12.58 ± 1.56 | 0.007 |  |
| (F) 2005-2014 (19-10) | 12.12 ± 1.33 | 12.16 ± 1.36 | 0.74 |  |
| Total | 12.62 ± 1.49 | 12.69 ± 1.49 | 0.17 |  |
| F-test (ANOVA) | ≤0.001 | ≤0.001 |  |  |
| Bonferroni’s post-hoc comparison (*p* -value)  A vs. B:  A vs. C:  A vs. D:  A vs. E:  A vs. F:  B vs. C:  B vs. D:  B vs. E:  B vs. F:  C vs. D:  C vs. E:  C vs. F:  D vs. E:  D vs. F:  E vs. F: | 1.0  1.0  0.034  ≤0.001  ≤0.001  1.0  0.42  ≤0.001  ≤0.001  0.19  ≤0.001  ≤0.001  0.003  1.0  ≤0.001 | 0.001  ≤0.001  ≤0.001  ≤0.001  ≤0.001  1.0  0.046  0.70  ≤0.001  1.0  1.0  0.007  1.0  0.41  0.029 |  |  |

Table S4 shows no significant association between season of birth and age at menarche. Although birth cohort had a significant effect, neither birth season nor the interaction between season and cohort was statistically significant.

**Table S4: Secular trend in mean age at menarche by season of birth**

| Age cohort | Birth season | | | | *p* -value | |
| --- | --- | --- | --- | --- | --- | --- |
|  | Winter (973,26.7%) | Spring  (936,25.7%) | Summer  (919,25.2%) | Autmn  (815, 22.4%) | F-test  (ANOVA) | Two-way ANOVA |
| (A) Before 1965 (≥60) | 13.14 ± 1.40 | 13.18 ± 1.23 | 13.04 ± 1.31 | 13.18 ± 1.46 | 0.78 | Birth season effect: F=2.23, *p* =0.08  Cohort effect: F=37.5, P≤0.001  Birth season x cohort interaction: F=0.45, *p* =0.97 |
| (B) 1965-1974 (59-50) | 12.92 ± 1.90 | 12.69 ± 1.62 | 12.86 ± 1.80 | 13.04 ± 1.75 | 0.40 |  |
| (C) 1975-1984 (49-40) | 12.82 ± 1.39 | 12.63 ± 1.31 | 12.94 ± 1.33 | 12.84 ± 1.26 | 0.26 |  |
| (D) 1985-1994 (39-30) | 12.65 ± 1.44 | 12.47 ± 1.36 | 12.57 ± 1.46 | 12.59 ± 1.54 | 0.71 |  |
| (E) 1995-2004 (29-20) | 12.39 ± 1.43 | 12.22 ± 1.61 | 12.37 ± 1.34 | 12.49 ± 1.49 | 0.45 |  |
| (F) 2005-2014 (19-10) | 12.22 ± 1.50 | 12.08 ± 1.13 | 12.13 ± 1.39 | 12.11 ± 1.32 | 0.81 |  |
| Total | 12.69 ± 1.54 | 12.53 ± 1.43 | 12.66 ± 1.48 | 12.69 ± 1.51 | 0.059 |  |
| F-test (ANOVA) | ≤0.001 | ≤0.001 | ≤0.001 | ≤0.001 |  |  |
| Bonferroni’s post-hoc comparison (*p* -value)  A vs. B:  A vs. C:  A vs. D:  A vs. E:  A vs. F:  B vs. C:  B vs. D:  B vs. E:  B vs. F:  C vs. D:  C vs. E:  C vs. F:  D vs. E:  D vs. F:  E vs. F: | 1.0  0.69  0.047  ≤0.001  ≤0.001  1.0  1.0  0.042  ≤0.001  1.0  0.19  0.005  1.0  0.15  1.0 | 0.032  0.015  ≤0.001  ≤0.001  ≤0.001  1.0  1.0  0.037  0.001  1.0  0.19  0.012  1.0  0.18  1.0 | 1.0  1.0  0.09  0.001  ≤0.001  1.0  1.0  0.048  ≤0.001  0.52  0.008  ≤0.001  1.0  0.17  1.0 | 1.0  0.98  0.016  0.003  ≤0.001  1.0  0.17  0.036  ≤0.001  1.0  0.75  ≤0.001  1.0  0.10  0.45 |  |  |

Table S5 demonstrates a modest association between birth order and age at menarche. Earlier menarche was observed among first-born or single girls compared with those of higher birth order. Significant effects of birth order and birth cohort were identified, whereas their interaction was not significant.

**Table S5: Secular trend in mean age at menarche by birth order**

| Age cohort | Birth order | | | | *p* -value | |
| --- | --- | --- | --- | --- | --- | --- |
|  | 1^st^/single  (1252,%34.4) | 2^nd^  (1031,28.3%) | 3^rd^  (720,19.8%) | 4^th^ & more  (640,17.6 %) | F-test  (ANOVA) | Two-way ANOVA |
| (A) Before 1965 (≥60) | 13.13 ±1.53 | 13.08 ± 1.32 | 13.21 ±1.14 | 13.12 ± 1.21 | 0.89 | Birth order effect: F=4.69, *p* =0.017  Cohort effect: F=20.94, *p* ≤0.001  Birth order xcohort interaction: F=1.64, *p* =0.056 |
| (B) 1965-1974 (59-50) | 12.66 ± 1.81a | 12.88 ± 1.80 | 12.96 ±1.64 | 13.21 ± 1.72  A | 0.058 |  |
| (C) 1975-1984 (49-40) | 12.76 ± 1.33 | 12.78 ± 1.40 | 12.84 ±1.13 | 12.93±1.41 | 0.74 |  |
| (D) 1985-1994 (39-30) | 12.34 ± 1.38a | 12.74 ± 1.48a | 12.72 ± 1.38 | 12.52 ±1.51 | 0.028 |  |
| (E) 1995-2004 (29-20) | 12.18 ± 1.50a | 12.23 ± 1.32b | 12.29 ± 1.47c | 12.92 ± 1.50  a,b,c | ≤0.001 |  |
| (F) 2005-2014 (19-10) | 11.99 ± 1.39 | 12.18 ± 1.32 | 12.18 ± 1.44 | 12.33 ±1.08 | 0.18 |  |
| Total | 12.52 ± 1.55a | 12.65 ± 1.48 | 12.68 ± 1.42 | 12.84 ± 1.46  a | ≤0.001 |  |
| F-test (ANOVA) | ≤0.001 | ≤0.001 | ≤0.001 | ≤0.001 |  |  |
| Bonferroni’s post-hoc comparison (*p* -value)  A vs. B:  A vs. C:  A vs. D:  A vs. E:  A vs. F:  B vs. C:  B vs. D:  B vs. E:  B vs. F:  C vs. D:  C vs. E:  C vs. F:  D vs. E:  D vs. F:  E vs. F: | 0.013  0.17  ≤0.001  ≤0.001  ≤0.001  1.0  0.43  0.02  ≤0.001  0.07  0.001  ≤0.001  1.0  0.31  1.0 | 1.0  0.77  0.41  ≤0.001  ≤0.001  1.0  1.0  0.001  ≤0.001  1.0  0.007  ≤0.001  0.017  0.004  1.0 | 1.0  0.68  0.1  ≤0.001  ≤0.001  1.0  1.0  0.004  ≤0.001  1.0  0.042  0.004  0.21  0.023  1.0 | 1.0  1.0  0.04  1.0  0.003  1.0  0.005  1.0  ≤0.001  0.48  1.0  0.047  0.45  1.0  0.04 |  |  |

a,b,c indicate significant pairwise differences between birth-order categories within the same birth cohort according to Bonferroni’s post-hoc multiple comparisons. The reported *p* -values for A–F comparisons represent Bonferroni-adjusted pairwise comparisons between birth cohorts within each birth-order category
